# Novel serum biomarkers modified by the body mass index z-score for the detection of liver fibrosis and steatosis in children with chronic hepatitis C

**DOI:** 10.1186/s12879-017-2462-1

**Published:** 2017-05-23

**Authors:** Maria Pokorska-Śpiewak, Barbara Kowalik-Mikołajewska, Małgorzata Aniszewska, Magdalena Pluta, Magdalena Marczyńska

**Affiliations:** 1 Department of Children’s Infectious Diseases, Medical University of Warsaw, ul. Wolska 37, 01-201 Warsaw, Poland; 2 Hospital of Infectious Diseases, ul. Wolska 37, 01-201 Warsaw, Poland

**Keywords:** Hepatitis C, Liver biopsy, Liver fibrosis, Liver steatosis

## Abstract

**Background:**

There is a need for validation of noninvasive alternatives to liver biopsy for the evaluation of fibrosis in children with chronic hepatitis C (CHC). The aim of this study was to evaluate the diagnostic performance of serum biomarkers modified by the body mass index z-score (BMI z-score) for the detection of fibrosis and steatosis in children with CHC.

**Methods:**

Thirty children aged 9.4 ± 3.7 years (14 males, 16 females) with CHC underwent liver biopsy. Fibrosis was scored using a 5-point METAVIR scale (≥2 = significant fibrosis). For all the children, the following noninvasive markers were calculated: The aspartate transaminase (AST)-to-platelets ratio index (APRI), the modified APRI (M-APRI: BMI z-score × APRI), the Fibrosis-4 index (FIB-4), the modified FIB-4 (M-FIB-4: BMI z-score × FIB-4), and a novel marker, B-AST (BMI z-score × AST). The area under the receiver operator characteristic curve (AUROC) was calculated to detect significant fibrosis and steatosis.

**Results:**

In the histopathological evaluation, 22/30 (73%) patients presented with fibrosis, and 8/30 (27%) presented with steatosis. For the detection of significant fibrosis, the AUROCs for M-APRI, M-FIB-4 and B-AST were 0.842, 0.823, and 0.848, respectively. For significant steatosis, the AUROCs were more than 0.9 for all markers that included the BMI z-score. B-AST, with a cut-off of 92.8, showed 71% sensitivity and 95% specificity for detecting significant fibrosis. For predicting severe steatosis, B-AST had 100% sensitivity and 92% specificity. Negative values of all three markers that included BMI z-scores excluded all patients with both significant fibrosis and significant steatosis.

**Conclusions:**

Including the BMI z-score in serum biomarker formulas enhances their diagnostic ability to detect significant fibrosis and steatosis. B-AST may thus act as an effective alternative to liver biopsy.

## Background

Liver biopsy has been considered a standard method for evaluating liver fibrosis and steatosis in children with chronic hepatitis C (CHC) [1–3]. However, it has several limitations: it is invasive and painful, has inter- and intraobserver variability, and is prone to sampling errors [1, 3]. Therefore, efforts have been made to develop alternative, noninvasive methods to liver biopsy, including imaging studies (elastography) and serum biomarkers [3, 4]. According to the recent recommendations of the European Association for the Study of the Liver (EASL), noninvasive methods can be used instead of liver biopsy to assess liver disease severity prior to antiretroviral therapy, and liver biopsy should be reserved only for cases with potential additional comorbidities or any uncertainty [2]. Many of these noninvasive methods have been evaluated in adults with CHC for their ability to determine fibrosis [5]. However, data on the accuracy of noninvasive tests in pediatric patients with HCV are limited, and none of these methods has been fully validated in children to date [3, 4].

The serum biomarkers include both direct and indirect markers. Direct markers (e.g.*,* glycoproteins, collagens, collagenases, and metalloproteases) reflect the removal or deposition of extracellular matrix in the liver [5]. Indirect markers can be identified in routine blood tests and indicate alterations in liver function [5]. Such markers include the aspartate transaminase-to-platelets ratio index (APRI) and the Fibrosis-4 index (FIB-4), which have been validated to predict significant fibrosis and cirrhosis in adult patients with chronic HCV infection [5–7]. Simple serum biomarkers are widely available, inexpensive, and easy to calculate and therefore provide a desirable alternative to liver biopsy [8].

In our recent studies, we have shown that in children with CHC, both fibrosis and steatosis were positively associated with the body mass index z-score (BMI z-score) [9, 10]. Thus, the aim of the present study was to analyze the diagnostic performance of APRI and FIB-4 to determine liver fibrosis and steatosis in children with CHC. Additionally, the diagnostic performance of these biomarkers when modified by including the BMI z-score in the formulas was established, and B-AST, a novel simple marker based on the BMI z-score and aspartate aminotransferase (AST) only, was proposed, and its diagnostic performance was analyzed.

## Methods

### Patients

This prospective clinicopathological study comprised consecutive treatment-naïve patients chronically infected with HCV who underwent a liver biopsy in our tertiary health care department between 2010 and 2014. Noninvasive serum biomarker analysis was performed simultaneously with liver biopsy as part of the qualification procedure for the antiviral treatment, according to the current European recommendations [2]. In addition, liver biopsy was required by the National Health Found for every patient prior to inclusion to the therapeutic program. The CHC diagnosis was established in patients with at least a 6-month history of hepatitis based on elevated alanine and aspartate aminotransferase (ALT and AST) serum levels and positive anti-HCV testing and was confirmed with nucleic acid testing/positive HCV RNA real-time polymerase chain reaction analysis (RT-PCR method; Amplicor, Roche; Cobas TaqMan, Roche). Both biochemical and serological testing were performed using commercially available laboratory kits (Vitros 5600, Ortho-Clinical Diagnostics, Johnson & Johnson). The upper limits of normal (ULN) for ALT and AST were established as 40 IU/l. The most likely route and time of infection were established using available medical records. The putative duration of infection was calculated from the beginning of the risk exposure.

Patients with hepatitis B virus, human immunodeficiency virus infection, autoimmune hepatitis, nonalcoholic fatty liver disease, Wilson’s disease, or alpha 1-antitrypsin deficiency were excluded from this study. The patients’ weights and heights were recorded on the day of the biomarker examination. BMI z-scores were calculated using the World Health Organization (WHO) Child Growth Standards and Growth Reference data with the WHO anthropometric calculator, AnthroPlus v.1.0.4.

### Biomarker evaluation

The biomarker determinations were performed using commercially available laboratory kits (XT-1800i, Sysmex for platelets; Vitros 5600, Ortho-Clinical Diagnostics, Johnson & Johnson for biochemical parameters). The noninvasive serum biomarker analysis included APRI and FIB-4, which were calculated according to the published analytic recommendations [6, 7], as follows:

$$ \begin{array}{l}\mathrm{APRI}=\left[\mathrm{AST}\left(\mathrm{IU}/\mathrm{L}\right)/\mathrm{AST}\;\mathrm{ULN}\left(\mathrm{IU}/\mathrm{L}\right)/\mathrm{platelet}\kern0.5em \mathrm{count}\left({10}^9/\mathrm{L}\right)\right]\times 100;\hfill \\ {}\mathrm{FIB}\hbox{-} 4=\left[\mathrm{age}\left(\mathrm{years}\right)\times \mathrm{AST}\left(\mathrm{IU}/\mathrm{L}\right)\right]/\left[\mathrm{platelet}\kern0.5em \mathrm{count}\left({10}^9/\mathrm{L}\right)\times \sqrt{\mathrm{ALT}\left(\mathrm{IU}/\mathrm{L}\right)}\right]\hfill \end{array} $$

Additionally, modified APRI (M-APRI) and modified FIB-4 (M-FIB-4) were calculated by including BMI z-scores in both formulas, as follows:

$$ \mathrm{M}\hbox{-} \mathrm{APRI}=\mathrm{BMI}\;\mathrm{z}\hbox{-} \mathrm{score}\times \mathrm{APRI};\mathrm{M}\hbox{-} \mathrm{FIB}\hbox{-} 4=\mathrm{BMI}\;\mathrm{z}\hbox{-} \mathrm{score}\times \mathrm{FIB}\hbox{-} 4. $$

A novel simple biomarker, B-AST = BMI z-score × AST (IU/L), was also proposed and calculated.

#### Histopathological evaluation

Liver biopsy was performed percutaneously using a Menghini needle (Hepafix kit 1.4 or 1.6 mm, Braun). An experienced pathologist who was unaware of the clinical data performed the histopathological evaluation. Fibrosis staging was evaluated using the METAVIR scoring system on a 5-point scale (F0 – no fibrosis; F1 – portal fibrosis without septa; F2 – portal fibrosis with few septa; F3 – numerous septa without cirrhosis; and F4 – cirrhosis) [11]. Fibrosis was considered significant if the METAVIR F score was ≥2. Liver steatosis was determined semi-quantitatively according to the percentage of hepatocytes containing fat droplets and was staged as follows: 0 – no steatosis; 1 – minimal (≤ 5% hepatocytes affected); 2 – mild (6–33%); 3 – moderate (34–66%); and 4 – severe (> 66%). Steatosis was considered significant if more than 33% of the hepatocytes were affected (steatosis score > 2).

### Statistical analysis

Continuous variables were tested for normal distribution using the Kolmogorov-Smirnov test and are expressed as the mean ± standard deviations (SD) or as medians with interquartile ranges (IQR), as required. To analyze associations between the results of the five serum biomarkers and fibrosis and steatosis stages, Spearman correlation coefficients were obtained. A two-sided *p*-value <0.05 was considered significant.

Receiver operating characteristic (ROC) curves and areas under the ROC curve (AUROC) calculations were performed to assess the diagnostic performances of the noninvasive tests for identifying patients with any or significant liver fibrosis and steatosis, using liver biopsy as a reference standard. An analyzed method is considered perfect when the AUROC is 100%, excellent when the AUROC is over 90%, and good when the AUROC is over 80% [3, 5]. Optimal cutoffs, sensitivity, specificity, and positive and negative predictive values were also calculated for each noninvasive test.

All statistical analyses were performed using the licensed MedCalc Statistical Software, ver. 17.2 (MedCalc, Mariakerke, Belgium).

### Ethical statement

The investigation was concordant with the principles outlined in the Declaration of Helsinki and its amendments. Written informed consent was collected from all the patients and/or their parents/guardians before their inclusion in the study.

## Results

### Patient characteristics

Thirty children (14 male and 16 female) aged 9.4 ± 3.7 years with a mean HCV infection duration of 8.2 ± 3.1 years were included in this study. Most of the children (73%) were infected vertically by an infected mother. The predominant HCV genotype in this group was 1b. In 2/30 (7%) cases, the BMI z-score indicated obesity (> 2 SD). The clinical and laboratory characteristics of the study group are presented in Table 1.

**Table 1:** Clinical and laboratory characteristics of the study group

Characteristics		Data
Number		30
Sex	Male (%)/female (%)	14 (47)/16 (53)
Age at liver biopsy (years)	Mean ± SD	9.4 ± 3.7
Duration of infection (years)	Mean ± SD	8.2 ± 3.1
BMI z-score	Mean ± SD	0.39 ± 1.04
Mode of infection	Vertical (%)	22 (73)
	Nosocomial (%)	7 (23)
HCV genotype (%)	1a	2 (7)
	1b	19 (63)
	3	5 (17)
	4	4 (13)
Viral load (IU/ml)	HCV median (IQR)	5.95 × 10^5^ (2.25 × 10^5^–1.53 × 10^6^)
Laboratory findings Mean ± SD/median (IQR)	ALT (IU/l)	60.5 (36–79)
	AST (IU/l)	50 (37–68)
	Platelets (× 10^9^/l)	308.2 ± 90.5
METAVIR F	0	8 (27)
	1	15 (50)
	2	7 (23)
	3–4	0
Steatosis	Any (%)	8 (27)
	Significant (%)	4 (13)
APRI	Mean ± SD	0.48 ± 0.26
FIB-4	Mean ± SD	0.22 ± 0.13
M-APRI	Mean ± SD	0.28 ± 0.69
M-FIB-4	Mean ± SD	0.09 ± 0.28
B-AST	Mean ± SD	31.71 ± 69.87

*ALT* alanine aminotransferase, *AST* aspartate aminotransferase, *BMI* body mass index, *APRI* aspartate-to-platelet Ratio Index, *FIB-4* Fibrosis-4 test, *M-APRI* modified aspartate-to-platelet ratio index (BMI z-score x APRI); *M-FIB-4* modified Fibrosis-4 index (BMI z-score x FIB-4), *B-AST* BMI z-score x AST

### Histopathological evaluation

Histopathological evaluation of the liver biopsy specimens revealed a mild stage of fibrosis in most cases: 22/30 (73%) patients presented with some stage of fibrosis, and in 7/30 (23%) patients, the fibrosis was significant (METAVIR F score ≥ 2 points). No cirrhosis cases were observed. Liver steatosis was detected in 8/30 (27%) patients, and in 4/30 (13%) patients, the steatosis was significant (> 33% of hepatocytes affected, Table 1).

### Noninvasive evaluation

Five noninvasive serum biomarkers were calculated according to the formulas described above, and the mean values for each test are presented in Table 1. The APRI exceeded 0.7 in 6/30 (20%) cases, and in 3/30 (10%) patients, the APRI was greater than 1.0. The maximal FIB-4 value was 0.57, and it did not exceed 1.0 in any case.

### Relationship between noninvasive and histopathological evaluations

Positive associations between the fibrosis stages determined by the histopathological evaluation and the M-APRI and B-AST scores were revealed (*p* = 0.01 and *p* = 0.001, respectively, Table 2). A trend toward such an association was observed for APRI and M-FIB-4 (*p* = 0.06 and *p* = 0.05, respectively, Table 2). Steatosis stages were positively associated with the APRI, M-APRI, and M-FIB-4 scores (*p* = 0.02 for all evaluations), and they trended toward an association with the B-AST score (*p* = 0.08, Table 2).

**Table 2:** Association between the five biomarkers and the stage of fibrosis (METAVIR F score) and between the biomarkers and the presence of steatosis

Marker	METAVIR F	Steatosis
APRI	0.34 (−0.03–0.63), *p* = 0.06	0.42 (0.08–0.68), *p* = 0.02
M-APRI	0.44 (0.08–0.69), *p* = 0.01	0.40 (0.05–0.67), *p* = 0.02
FIB-4	0.18 (−0.20–0.51), *p* = 0.35	0.09 (−0.27–0.44), *p* = 0.61
M-FIB-4	0.36 (−0.01–0.64), *p* = 0.05	0.40 (0.04–0.66), *p* = 0.02
B-AST	0.56 (0.24–0.77), *p* = 0.001	0.32 (−0.05–0.61), *p* = 0.08

Data are presented as correlation coefficients (95% confidence intervals) and *p*-values

**Fig. 1:**
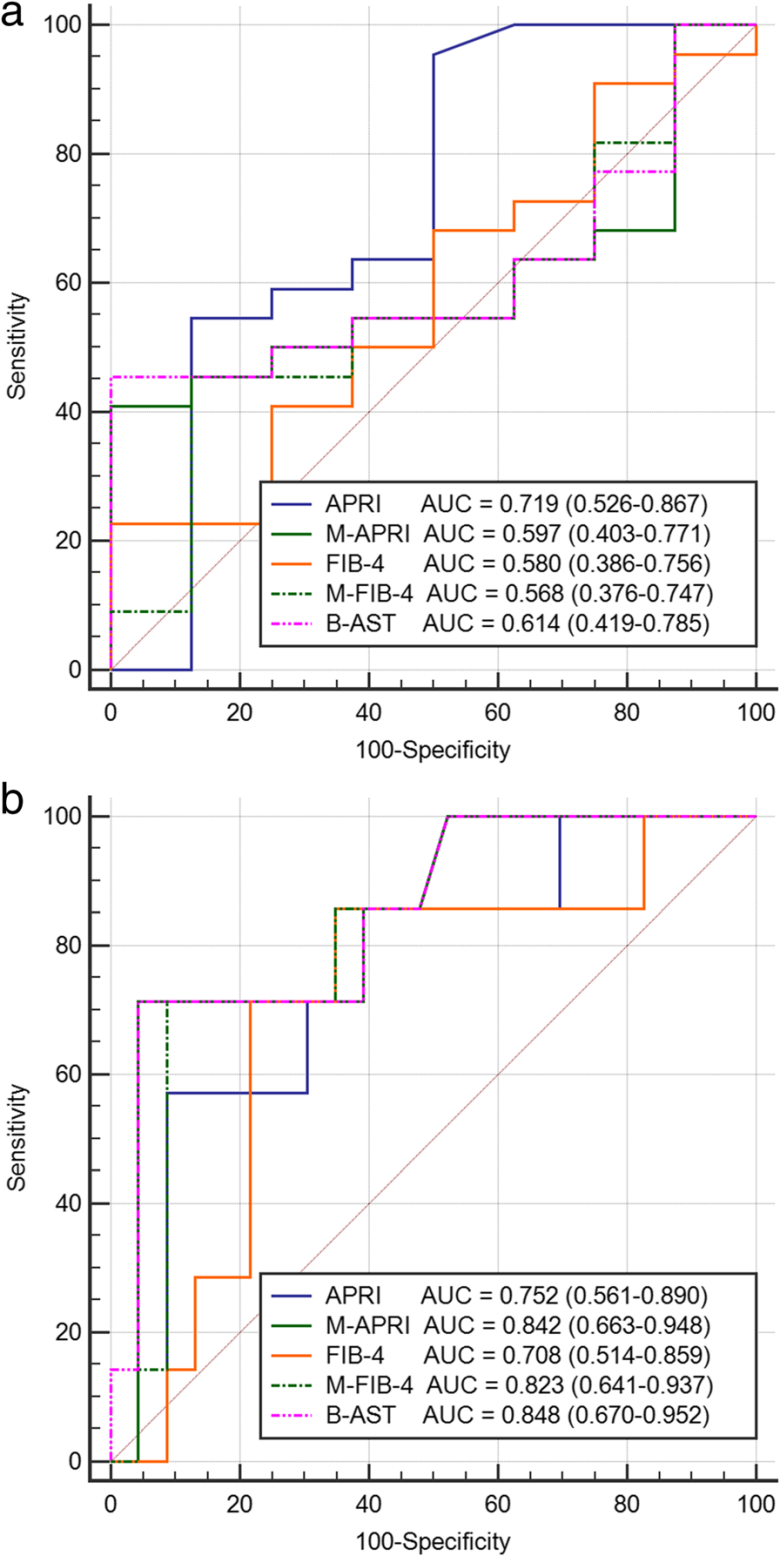
Receiver operating characteristic (ROC) curves of the five noninvasive liver fibrosis tests for detecting fibrosis. **a** for fibrosis at any stage (METAVIR F score > 0 points). **b** for significant fibrosis (METAVIR F score ≥ 2 points)

Figure 1 shows the diagnostic performance (ROC curves) of the five analyzed noninvasive tests for all fibrosis diagnoses (Fig. 1a) and for significant fibrosis diagnoses (METAVIR F ≥ 2 points, Fig. 1b). For the diagnosis of any fibrosis stage, the corresponding AUROCs were below 0.8 for all tests, and the highest AUROC value was observed for APRI: 0.719 (0.526–0.867) (Fig. 1a). In the significant fibrosis cases, the AUROCs for all three tests that included BMI z-scores exceeded 0.8 (0.842, 0.823, and 0.848 for the M-APRI, M-FIB-4, and B-AST, respectively), whereas the AUROCs for the APRI and FIB-4 were below 0.8 (Table 3). The novel marker, B-AST, predicted significant fibrosis with a cut-off of 92.8 with 71.4% sensitivity and 95.7% specificity.

**Table 3:** Diagnostic performance of five non-invasive tests for determining significant fibrosis (METAVIR F ≥ 2)

Significant fibrosis (METAVIR F ≥ 2)					
Test	APRI	M-APRI	FIB-4	M-FIB-4	B-AST
AUROC (95% CI)	0.752 (0.561–0.890)	0.842 (0.663–0.948)	0.708 (0.514–0.859)	0.823 (0.641–0.937)	0.848 (0.670–0.952)
Cut-off	0.656	0.577	0.180	0.179	92.82
Sensitivity (95% CI)	57.1 (18.4–90.1)	71.4 (29.0–96.3)	85.7 (42.1–99.6)	71.4 (0.29–96.3)	71.4 (0.29–96.3)
Specificity (95% CI)	91.3 (72.0–98.9)	95.6 (78.1–99.9)	65.2 (42.7–83.6)	91.30 (72.0–98.9)	95.7 (78.1–99.0)
+ PV	66.7 (31.5–89.7)	83.3 (41.0–97.3)	42.9 (28.4–58.6)	71.4 (38.0–91.1)	83.3 (41.0–97.3)
- PV	87.5 (74.7–94.3)	91.7 (77.3–97.3)	93.7 (70.5–99.0)	91.3 (76.4–97.2)	91.7 (77.3–97.3)

*APRI* aspartate-to-platelet ratio index, *FIB-4* Fibrosis-4 index, *M-APRI* modified aspartate-to-platelet ratio index (BMI z-score x APRI), *M-FIB-4* modified Fibrosis-4 index (BMI z-score x FIB-4), *B-AST* BMI z-score x AST, *AUROC* area under the receiver operating characteristic, *95% CI* 95% confidence interval, *+ PV* positive predictive value, *− PV* negative predictive value

**Fig. 2:**
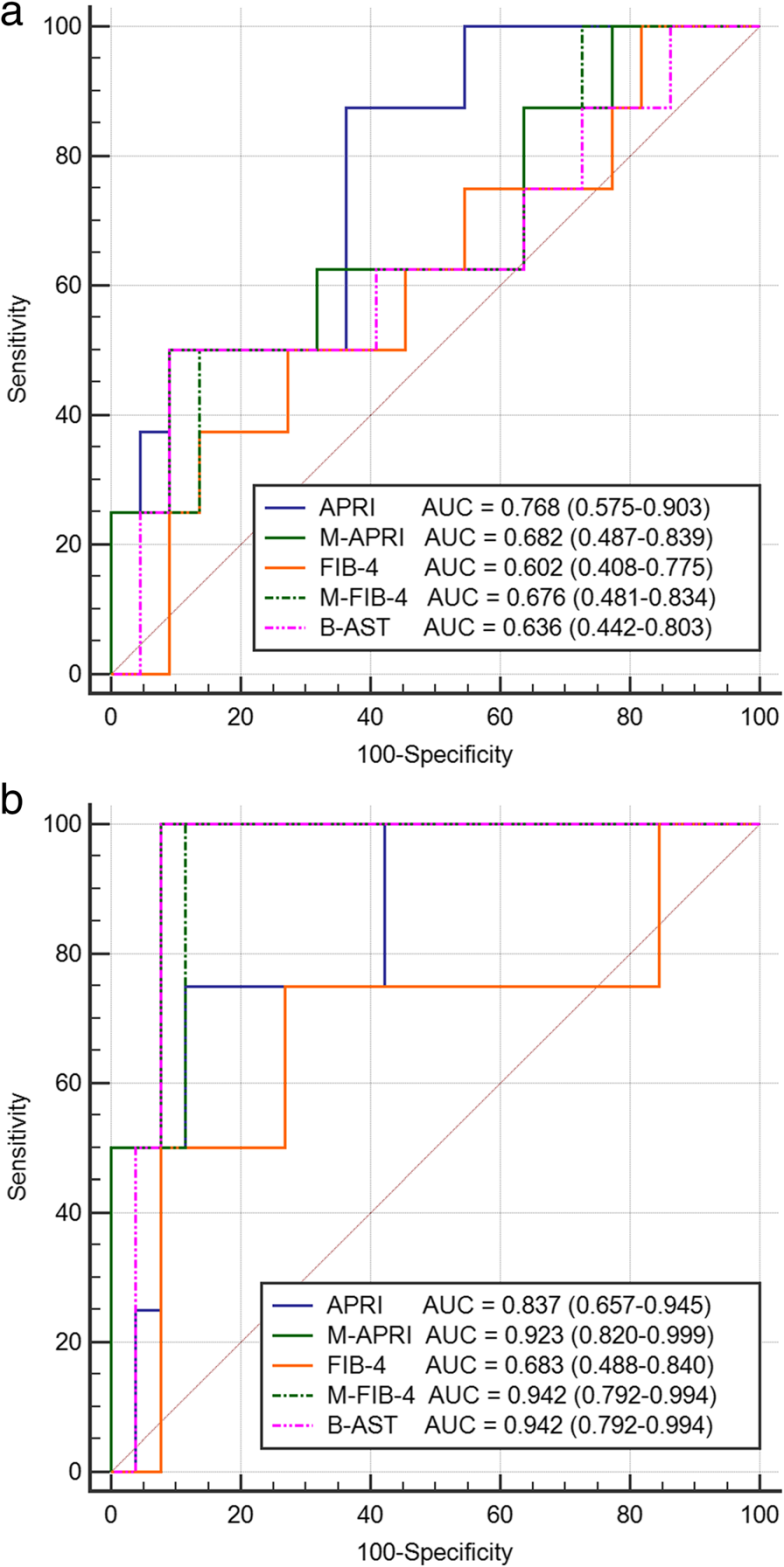
Receiver operating characteristic (ROC) curves of the five noninvasive liver fibrosis tests for detecting steatosis. **a** for any steatosis. **b** for significant steatosis (> 33% of hepatocytes affected)

The diagnostic performances of the five noninvasive tests for determining steatosis are presented in Fig. 2 and Table 4. For the detection of any steatosis stage, the AUROC was highest for the APRI (0.768). The AUROCs for the other tests did not exceed 0.7 (Fig. 2a). The diagnostic value of the tests for detecting significant steatosis was excellent (AUROC >0.9) for all three tests that included the BMI z-scores (0.923, 0.942, and 0.942 for M-APRI, M-FIB-4, and B-AST, respectively). For the APRI, the AUROC was good (0.837); however, for the FIB-4, the AUROC was insufficient (0.683). B-AST, with a cut-off of 92.8, predicted significant steatosis with 100% sensitivity and 92.3% specificity (Table 4).

**Table 4:** Diagnostic performance of five non-invasive tests for determining significant steatosis (> 33% of hepatocytes)

Significant steatosis (> 33% of hepatocytes)					
Test	APRI	M-APRI	FIB-4	M-FIB-4	B-AST
AUROC (95% CI)	0.837 (0.657–0.945)	0.923 (0.820–0.999)	0.683 (0.488–0.840)	0.942 (0.792–0.994)	0.942 (0.792–0.994)
Cut-off	0.656	0.577	0.216	0.179	92.82
Sensitivity (95% CI)	75 (19.4–99.4)	100 (39.8–100)	75 (19.4–99.4)	100 (39.8–100)	100 (39.8–100)
Specificity (95% CI)	88.46 (69.8–97.6)	92.3 (74.9–99.1)	73.1 (52.2–88.4)	88.46 (69.8–97.6)	92.3 (74.9–99.1)
+ PV	50.0 (23.1–76.9)	66.7 (34.6–88.3)	30.0 (15.5–50.0)	57.1 (31.5–79.4)	66.7 (34.6–88.3)
- PV	95.8 (80.7–99.2)	100	95.0 (77.4–99.1)	100	100

*APRI* aspartate-to-platelet ratio index, *FIB-4* Fibrosis-4 index, *M-APRI* modified aspartate-to-platelet ratio index (BMI z-score x APRI), *M-FIB-4* modified Fibrosis-4 index (BMI z-score x FIB-4), *B-AST* BMI z-score x AST, *AUROC* area under the receiver operating characteristic, *95% CI* 95% confidence interval, *+ PV* positive predictive value, *− PV* negative predictive value

## Discussion

In the era of direct-acting antivirals (DAAs), successful treatment of CHC is achievable for nearly all infected patients, and DAAs are recommended for all adults with chronic HCV infection [2, 12]. The availability of DAAs for pediatric patients with CHC is expected within the coming months. According to the current recommendations, histopathological evaluation of liver disease may not be necessary for all patients prior to therapy. However, the majority of patients require testing to determine the stage of fibrosis, which is vital for determining the urgency of treatment, the duration of treatment in some instances, and the need for more intensive clinical monitoring [12].

Several noninvasive markers of liver fibrosis based on a combination of different biochemical parameters have been developed recently and analyzed in adult patients with CHC [5, 7, 8, 13]. A meta-analysis of 40 studies revealed that an APRI score greater than 1.0 had a sensitivity of 76% and specificity of 72% for predicting cirrhosis. Additionally, APRI scores greater than 0.7 had a sensitivity of 77% and specificity of 72% for predicting significant hepatic fibrosis [13]. In a study by Sterling et al., FIB-4 scores <1.45 had a negative predictive value of 90% for advanced fibrosis, and FIB-4 scores >3.25 had a 97% specificity and a positive predictive value of 65% for advanced fibrosis [6].

There are only limited published data on the use of these noninvasive methods in children, and, to our knowledge, none of these serum biomarkers have been fully validated in children with CHC to date. De Ledinghen et al. prospectively analyzed the feasibility of a liver stiffness measurement using FibroScan (elastography method) in 116 children with chronic liver diseases and compared the results with those of FibroTest (a commercial biomarker), the APRI and liver biopsy [4]. All three noninvasive methods correlated significantly with the METAVIR fibrosis score. The AUROCs for cirrhosis diagnoses were 0.88 for FibroScan, 0.73 for FibroTest, and 0.73 for the APRI. However, the cohort of patients in this study was heterogenic and included children with liver diseases of different etiologies.

In a study conducted in children and adolescents with chronic hepatitis B and C, the AUROC was 0.71 for identifying patients with fibrosis and 0.52 for identifying those with liver cirrhosis [14]. For children infected with HCV, the AUROC was 0.75; it was more effective for vertically infected patients than for those infected via blood transfusion (1.00 vs. 0.53). The authors concluded that a validated noninvasive marker of fibrosis is needed for pediatric patients; however, the results of their study indicated that the APRI is not such a marker [14].

Our recent observations suggest that liver fibrosis in children with CHC is positively associated with the BMI z-score. In both univariate and multivariate analyses, the BMI z-score was found to be an independent predictor of fibrosis among our 42 pediatric patients with CHC (*p* = 0.03) [9]. Therefore, we proposed including the BMI z-score in the formulas of previously used biomarkers. The obtained results indicate that these modified tests perform better than the APRI and FIB-4 for predicting severe fibrosis. Additionally, we proposed a new simple biomarker, B-AST, that can easily detect liver fibrosis based on very simple parameters. B-AST, with a cut-off of 92.8, showed 71% sensitivity and 95% specificity for detecting significant fibrosis.

Several studies have demonstrated that liver steatosis in patients with CHC may have prognostic and metabolic implications [15–17]. In our recent study, moderate to severe steatosis was independently associated with the BMI z-score in a group of 48 patients with CHC (*p* = 0.02) [10]. In addition, it was found to be a predictor of advanced fibrosis in children with HCV infection; this was a unique finding because unlike in adults, no correlation between liver steatosis and fibrosis had previously been confirmed in pediatric patients with CHC [10]. Noninvasive markers of steatosis have been studied in patients with nonalcoholic fatty liver disease (NAFLD) but not in patients with CHC [18]. The results of our study suggest that biomarkers that include the BMI z-score perform excellently for diagnosing significant steatosis in children with CHC. B-AST had a very high sensitivity (100%) and specificity (92%) for predicting severe steatosis with a cut-off of 92.8. Considering that negative B-AST values excluded all patients with both significant fibrosis and significant steatosis, one may speculate that liver biopsy could be avoided in children with B-AST <0. In this study, 11/30 (37%) of children had B-AST <0 and consequently would not require the liver biopsy.

Despite the novel and unique findings presented in this study, several issues should be considered as limitations. First, the small number of patients in the study group should be acknowledged, and the obtained results should be confirmed with other, larger cohorts of children. However, currently, liver biopsy is rarely performed in children; thus, the opportunity to compare noninvasive methods with histopathological assessment as a reference standard is lacking. Another important issue arose from the relatively mild liver disease observed in the histopathological evaluations: a low number of children presented with significant fibrosis, and no children presented with cirrhosis, which could lead to a spectrum bias. However, children with CHC usually present with mild liver disease, and cirrhosis is rarely observed [9]. Therefore, in most cases, distinguishing no or mild fibrosis from significant fibrosis would be most desirable for therapeutic decision-making.

## Conclusions

In conclusion, the results of this study indicate that noninvasive biomarkers that include BMI z-scores in their formulas have a good to excellent performance for detecting significant liver disease. The novel simple B-AST serum test may be an inexpensive and widely available alternative to liver biopsy due to its high sensitivity and specificity for detecting significant liver fibrosis and steatosis. Continued research in this area in pediatric populations is needed to fully validate these noninvasive diagnostic tools for children with chronic liver diseases of different etiologies.

## Abbreviations

ALT: Alanine aminotransferase
APRI: Aspartate aminotransferase-to-platelet ratio index
AST: Aspartate aminotransferase
AUROC: Area under the receiver operating characteristic
BMI z-score: Body mass index z-score
CHC: Chronic hepatitis C
CI: Confidence interval
HCV: Hepatitis C virus
IQR: Interquartile ranges
PV: Predictive value
ROC: Receiver operating characteristic
SD: Standard deviation
ULN: Upper limit of normal
